# High Prevalence of Poststroke Depression in Ischemic Stroke Patients in Ethiopia

**DOI:** 10.1155/2020/8834299

**Published:** 2020-10-29

**Authors:** Fikru Tsehayneh, Abenet Tafesse

**Affiliations:** Department of Neurology, Addis Ababa University School of Medicine, Addis Ababa, Ethiopia

## Abstract

**Background:**

There is limited information and research carried out regarding the prevalence of poststroke depression (PSD) in the study area. Psychiatric disorders complicate a significant proportion of patients suffering from stroke. This of course have a great negative impact on our knowledge about poststroke depression in Ethiopia, and poststroke depression complicates a significant number of stroke patients and their rehabilitation.

**Methods:**

A cross-sectional study on all patients aged above 18 years and diagnosed with stroke in the past two years who attended the neurology follow-up clinics of Tikur Anbessa Specialized Hospital (TASH) and Zewditu Memorial Hospital (ZMH) was done by using a structured questionnaire containing Patient Health Questionnaire-9 (PHQ-9) depression screening tool.

**Result:**

Of 84 patients who were eligible for the study, 32.2% of patients have depressive symptoms. Women (OR 0.001, 95% CI 0.12–0.87) and aphasic patients (OR 0.034, 95% CI 0.19–1.27) were more likely to have depressive symptoms.

**Conclusion:**

Depressive symptoms after stroke are common in Ethiopian patients. Our study demonstrates female and aphasic patients are more likely to screen positive for PSD. Hence, screening all poststroke patients with different screening tools is practical, and further studies are needed to assess the validity of these screening tools and also to assess PSD as a predictor of stroke outcome.

## 1. Background

Stroke is becoming a leading cause of death and adult disability in the developing world. It is the second leading cause of death worldwide comprising about 10% of all the deaths which is 5.5 million people per year and with 44 million disability-adjusted life years (DALYs) lost. The global burden of disease study indicates 16.9 million stroke cases in 2010 and 70% occurred in low- and middle-income countries [1].

In Ethiopia, stroke is a common cause of mortality and morbidity. It is the most common neurological disorder seen in patients admitted to general hospitals in Ethiopia [2]. Cerebrovascular disease (CVD) was reported to account for 23.6% of all neurological admissions in Tikur Anbessa Specialized Hospital (TASH) [3].

Psychiatric disorders are known to complicate a significant proportion of patients in the poststroke period. In the acute phase of stroke, delirium and other neuropsychiatric complications become a focus of interest as they disturb the process of care and are indicators of worse outcome. Psychiatric disturbances strongly contribute to a low quality of life among stroke survivors in the long term [4].

A study showed that nonpsychiatric physicians fail to diagnose 50% to 80% of the actually existing depression. Poststroke depression has been related to physical disability and the limitations in daily activities. However, PSD is most likely not solely a psychological reflection of a physical disability. Another study showed that stroke patients were significantly more commonly depressed than orthopedic patients with equal levels of functional disability. It has been claimed that PSD is related to specific locations of ischemic brain lesions, although this has also been denied [5, 6].

Different screening tools for use in screening depression were used in previous literature studies. Evidence suggests that the Patient Health Questionnaire-9 (PHQ-9) performs well as a brief screen for poststroke depression and has a greater sensitivity and specificity for depression [7]. The PHQ-9 is a multipurpose instrument for screening, diagnosing, monitoring, and measuring the severity of depression. It is brief and useful in clinical practice, and it can also be administered repeatedly, which can show improvement or worsening of depression in response to treatment [8].

Significant proportions of aphasic patients (62–70%) were found to have depression possibly due to loss of communication with their friends and difficulty engaging in social activities [9].

Clinically significant depression is frequent after ischemic stroke. And emphasis should be given to the importance of the psychiatric examination of poststroke patients, especially those with a significant disability and with a history of prior depressive episodes [5].

It is likely that depression reduces the desire and ability to take part in rehabilitation, and depressed people are less inclined to socialize. Low social support is known to be associated with depression. Other social factors associated with depression in poststroke period are living alone or in an institution. This social isolation may be complicated by the stigma associated with mental health problems, with some people hiding symptoms so that their family and doctor are unaware of any problems. Poor adherence to medications and increased alcohol and drug intake are commonly observed in depressed and socially isolated individuals. In turn, loss of physical ability and decreased cognitive function after stroke may also lead to depression [10].

A study assessing the adequacy of SSRI treatment in PSD patients found out that those patients who responded well to the treatment has shown a better functional outcome than nonresponders to SSRI treatment. Poststroke depression remains an unfavorable factor in rehabilitating poststroke patients despite treatment with antidepressants [11]. Another study assessed the role of improvement of depression in social role functioning. Social role function is often termed as participation in the rehabilitation sciences literature. Participation is broadly defined by the World Health Organization's International Classification of Functioning, Disability and Health (ICF) 7 as a person's involvement in a life situation. It represents the societal perspective of functioning. The study found out that improvement of depression was independently associated with improvement in the social role function of patients which positively affects the rehabilitation of patients [12].

Significantly increased functional disability and poorer quality of life at 1 month poststroke was seen in patients with acute PSD after controlling for relevant confounders [13]. In another prospective study, depression detected 3 months after stroke has been independently associated with poor functional outcome [14]. These findings are not consistent, and another study showed the severity of depressive symptoms was not found to be independent predictor of length of survival in the short- and long-term follow-up of 18 years [15].

## 2. Method

A cross-sectional study on all patients aged above 18 years and diagnosed with stroke in the past two years who attended the neurology follow-up clinics of Tikur Anebessa specialized Hospital and Zewditu Memorial Hospital was done by using a structured questionnaire after obtaining informed consent.

Patients with diagnosis other than stroke, stroke patients who presented to the clinics after 2 years of sustaining stroke, and those with severe aphasia were excluded from the study.

Different variables including age, sex, marital status, ethnicity, educational level, occupation, religion, aphasia, type of stroke, hemisphere affected on imaging, previous psychiatric illness, and PHQ-9 score were assessed. PHQ-9 is a multipurpose instrument for screening, diagnosis, monitoring, and measuring the severity of depression. It assesses 9 parameters each scored 0–3 (0 = not at all, 1 = several days, 2 = more than half the days, and 4 = nearly every day). A sum of all these scores form the basis for the scale score that ranged between zero and twenty seven (score 0–4 = no depression, score of 5–9 = minimal symptoms, score of 10–14 = minor depression, score of 15–19 = moderately severe depression, and score of 20–27 = severe depression). The PHQ-9 questionnaire was translated into Amharic (the Ethiopian local language) and back translated into English to ensure quality of translation.

Descriptive statistics for all patients, as well as stratified per stroke type, are presented as frequencies for nominal and categorical data and as means ± standard deviation or median with interquartile range for continuous variables. Proportions with 95% confidence intervals have been reported for feasibility metrics. The PHQ-9 scale and extremity power were dichotomized for certain analyses (PHQ-9 > 10 as indication of depression). Univariable and multivariable logistic regression were fit to determine factors associated with PHQ-9 screening and for screening positive with the PHQ-9. Linear regression models were fit to explore association of continuous PHQ-9 score with demographic and clinical variables. In both logistic and linear models, important interactions were also assessed. All statistical tests were performed at 5% level of significance using SPSS/PC version 20.0 software packages for statistical analysis (SPSS, INC, Chicago, IL).

## 3. Results

### 3.1. Characteristics of the Participants

Eighty four patients who came for follow-up at Tikur Anbessa Specialized Hospital neurology clinic and Zewditu Memorial Hospital with stroke which occurred in the past two years were enrolled consecutively into our study and analyzed. Stroke type was confirmed by head CT scan and MRI, and out of this, 61 (72.6%) had an ischemic stroke, and 23 (27.4%) had haemorrhagic stroke.

Age range was 20 to 90 years with mean age ± SD, overall was 57.1 ± 14.8 years, 52.4% were male, and 47.6% were female. Baseline sociodemographic profile of the study subjects is shown in Table 1.

Fifty seven (67.9%) of the study participants have left hemisphere affection. And 34 (40.5%) of participants have aphasia. All our patients with aphasia were able to understand the questionnaire as they were evaluated during the follow-up outpatient visit, and the language deficits were resolving.

### 3.2. Factors Associated with Poststroke Depression

**Figure 1:**
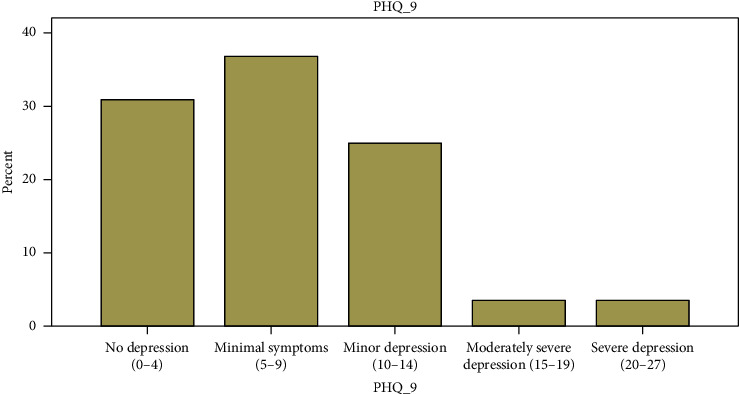
Frequency distribution of depression among stroke patients at TASH and ZMH neurology clinics.

Overall, almost one-third of all ischemic stroke and hemorrhagic stroke patients had depressive symptoms, as defined in our study as PHQ-9 score ≥10. Twenty seven patients out of 84 (32.2%) had depressive symptoms. Six patients' had moderate to severe depressive symptoms (Figure 1).

A majority of patients with depression are aged greater than 55 years, and two of the severely depressed patients are found in this age group as shown in Table 2. Fifteen out of twenty one patients with minor depression have left hemisphere affection on imaging (Table 3).

In both univariable and multivariable models, female sex (OR 0.97, 95% CI 0.96–0.99) and aphasia (OR 2.06, 95% CI 1.06–4.01) were associated with screening positive for PSD (Table 4). When depression score was treated as a continuous variable, none of the factor was associated with screening positive for PSD.

## 4. Discussion

The overall rate of depression in our subset of ischemic and hemorrhagic stroke patients was 32.2%, comparable to previously published data for depression in the stroke population as a whole [4, 8, 11, 16]. Fifty seven out of eighty four patients (67.8%) have unremarkable depressive symptoms.

In our study, females and aphasic patients were more likely to screen positive for depression. In accordance with our study, among United States adults, females have higher depression prevalence rates compared to males [17]. It is possible that an underlying, sex-specific predisposition to depression in females and the effect of sex hormones persists in the stroke patients as well.

In our study, 40.5% of patients screened positive for depression, and literature studies show a high prevalence of depression (62–70%) in patients with aphasia because of difficulty in communication with their friends and family members. Sixty seven (67.9%) of patients had left hemisphere affection, out of which 20 (35%) screened positive for depression, and other studies also showed that individuals with left hemisphere lesions may be particularly at a greater risk of developing depression and anxiety after stroke [18].

Out of the 84 patients enrolled in the study, 61 (72%) patients had ischemic stroke, and the rest had hemorrhagic stroke. And 21 (34.5%) of ischemic stroke patients screened positive for depression, and 6 (26%) of patients with hemorrhagic stroke also screened positive for depression. Clinically significant depression is frequent after ischemic stroke [5, 6].

Due to resource constraints, the study was limited to a small number of stroke cases on follow-up, and hence it is difficult to generalize the findings to the general stroke population. Other limitations of the study are shortage of research studies done on the topic in the study area, and comorbid psychiatric symptoms were not evaluated and may also have been common in this population.

Due to absence of a depression-control group without stroke, it is difficult to determine the degree to which the pattern of depressive symptoms discerned in this population is directly related to comorbid stroke. Other depression screening tools in aphasic patients in addition to the PHQ-9 screening tool may provide a more reliable result regarding depressive symptoms in this specific patient population.

## 5. Conclusion

In conclusion, depressive symptoms after stroke are common in Ethiopian patients as seen in the other parts of the world. Identification and treatment of PSD may positively contribute for patient recovery. Our study demonstrates female and aphasic patients are more likely to screen positive for PSD. Hence, screening all poststroke patients with different screening tools is practical, and further studies are needed to assess the validity of these screening tools and also to assess PSD as a predictor of stroke outcome.

## Figures and Tables

**Table 1:** Baseline sociodemographic characteristics of stroke patients at TASH and ZMH neurology clinic.

	Variables and categories	Frequency *N* = 84	Percentage (%)
Gender	Female	40	47.6
	Male	44	52.4

Age group	15–24	2	2.4
	25–34	4	4.8
	35–44	10	11.9
	45–54	17	20.2
	55–64	24	28.6
	>65	27	32.1

Marital status	Unmarried	24	28.6
	Married	60	71.4

Education	No formal education	30	35.7
	Secondary school	30	35.7
	More than secondary	24	28.6

**Table 2:** Frequency distribution of depression with age group among stroke patients at TASH and ZMH neurology clinics.

		Depression					Total
		No depression (0–4)	Minimal symptoms (5–9)	Minor depression (10–14)	Moderately severe depression (15–19)	Severe depression (20–27)	
Age group	15–24	0	2	0	0	0	2
	25–34	0	3	1	0	0	4
	35–44	4	2	4	0	0	10
	45–54	6	8	2	0	1	17
	55–64	9	7	7	1	0	24
	>65	7	9	7	2	2	27
Total		26	31	21	3	3	84

**Table 3:** Frequency distribution of depression with hemisphere affected on imaging among stroke patients at TASH and ZMH neurology clinics.

		Depression					Total
		No depression (0–4)	Minimal symptoms (5–9)	Minor depression (10–14)	Moderately severe depression (15–19)	Severe depression (20–27)	
Hemisphere affected on imaging	Left	17	20	15	3	2	57
	Right	8	7	5	0	0	20
	Both	1	4	1	0	1	7
Total		26	31	21	3	3	84

**Table 4:** Factors associated with screening positive for poststroke depression imaging among stroke patients at TASH and ZMH neurology clinics.

Variable		*p* value	Crude OR (95% CI)	*p* value	Adjusted OR (95% CI)
**Handedness**					
Right		1.00	1.00	0.99	0.00 (0.00–0.0)
Left		0.99	0.00 (0.0–0.0)		

**Gender**					
Male		1.00	1.00	0.001	10.21 (2.43–42.78)
Female		0.26	0.33 (0.12–0.87)		

**Age group (years)**					
15–24		0.99	0.00 (0.00–0.00)	0.54	1.15 (0.73–1.82)
25–34		0.55	0.48 (0.04–5.29)		
35–44		0.976	0.97 (0.22–4.26)		
45–54		0.12	0.31 (0.07–1.35)		
55–65		0.58	0.73 (0.23–2.28)		
65 and above		1.00	1.00		

**Marital status**					
Couples in union		1.00	1.00	0.95	0.96 (0.28–3.37)
Not in union (separated/divorced, widowed, and never married)		0.84	0.90 (0.32–2.53)		

**Duration of stroke**					
≤ 3 months		1.00	1.00	0.66	1.17 (0.59–2.33)
≤ 6 months		0.66	1.33 (0.37–4.83)		
≤ 2 years		0.97	1.03 (0.34–3.09)		

**Employment**					
Employed		1.00	1.00	0.89	0.92 (0.28–3.02)
Nonemployed		0.68	0.82 (0.32–2.07)		

**Educational level**					
No formal		1.00	1.00	0.17	0.69 (0.42–1.16)
Primary		1.0	1.00 (0.29–3.45)		
Secondary		0.6	0.67 (0.15–3.02)		
More than secondary		1.00	1.00 (0.32–3.12)		

**Type of stroke**					
Ischemic		1.00	1.00	0.97	1.03 (0.28–3.81)
Hemorrhagic		0.47	0.67 (0.23–1.96)		

**Hemisphere affected**					
Left		1.00	1.00	0.56	0.76 (0.3–1.94)
Right		0.41	0.62 (0.19–1.95)		
Both		0.73	0.74 (0.13–4.16)		

**Aphasia**					
Yes		1.00	1.00	0.034	0.25 (0.07–0.90)
No		0.15	0.50 (0.19–1.27)		

**Extremity power change**					
Improved		1.00	1.00	0.57	0.71 (0.22–2.31)
No improvement		0.55	1.33 (0.52–3.4)		

**Previous psychiatric**				
Yes		0.99	0.00 (0.00)	0.99	0.00 (0.0)
No		1.00	1.00		

## Data Availability

The datasets used and/or analyzed during the current study are available from the corresponding author on reasonable request.
